# What distinguishes life satisfaction from emotional wellbeing?

**DOI:** 10.3389/fpsyg.2024.1434373

**Published:** 2024-09-27

**Authors:** Filip Fors Connolly, Tommy Gärling

**Affiliations:** ^1^Department of Sociology, Umeå University, Umeå, Sweden; ^2^Department of Psychology, University of Gothenburg, Gothenburg, Västergötland, Sweden

**Keywords:** antecedent factors, emotional wellbeing, life satisfaction, survey, Swedish citizens, subjective well-being

## Abstract

The aim of this registered report is to investigate how the core components of subjective wellbeing, Life Satisfaction (LS) and Emotional Wellbeing (EWB), differ with respect to their relationship to antecedent factors. Seven factors are proposed that have been found in previous research to antecede LS and EWB. Social Comparisons, Meaningfulness, Economic/Social Resources, and Opportunities are hypothesized to correlate more closely with LS than EWB, while Time Use, Hassles/Uplifts, and Neuroticism are hypothesized to correlate more strongly with EWB than LS. A combined online and paper-and-pencil survey was administered to a representative sample of 974 Swedish citizens aged 15 years or older. LS and EWB were measured by self-report methods that have been developed and used in previous research. Index measures were constructed from self-reports of each antecedent factor by means of ratings on two scales developed in this study. Multivariate regression and relative weight analyses confirmed two of the hypotheses in showing that Economic/Social Resources and Opportunities were significantly more strongly related with LS than EWB. Of those hypothesized to correlate more strongly with EWB than LS, support was obtained for Neuroticism. Social Comparisons, Meaningfulness, Time Use, and Hassles/Uplifts did not have different relationships with LS and EWB. The results confirm that to some extent antecedent factors are differentially associated with LS and EWB, thus contributing to a better understanding of the antecedents of the cognitive and affective components of subjective wellbeing. Future research should further explore the mechanisms underlying these different relationships as well as moderators and mediators of the relationships.

## 1 Introduction

Subjective wellbeing (SWB) is generally proposed to include two primary components: Life Satisfaction (LS) and Emotional Wellbeing (EWB) (Diener, 1994; Tov, 2018; Busseri and Sadava, 2011). LS refers to cognitive judgments of overall life satisfaction; EWB refers to the balance between the frequency in daily life of experiences of positive and negative affect. Busseri and Quoidbach (2022) showed that SWB may be conceptualized as a higher-order latent factor with LS, positive affect (PA), and negative affect (NA) as indicators. While this is useful for some purposes (see e.g., Busseri and Erb, 2023), a substantial body of research emphasizes the distinction between LS and EWB, both with respect to theoretical conceptions of SWB and empirical outcomes (Diener, 1994; Brülde, 2007; Kahneman, 2011; Fors Connolly and Gärling, 2023). This is recognized by Busseri and Quoidbach who note that “understanding individuals' momentary experiences of SWB requires consideration of, and attention to, both the shared and unique aspects of LS, PA, and NA” (p. 10).

The relationship between LS and EWB is generally positive, with correlation coefficients typically ranging from moderate to strong (*r*s ranging from 0.40 to 0.90; see Berlin and Fors Connolly, 2019). Even if the correlation is strong, each component accounts for a significant amount of unique variance. Busseri and Erb (2023) found this to be true even after controlling for the relationships with the Big Five personality traits. It has accordingly been shown in several studies that the two components of SWB are also associated with other factors than personality traits (e.g., Kahneman and Krueger, 2006; Knabe et al., 2010; Fors Connolly et al., 2021; Fors Connolly and Gärling, 2023). For instance, Luhmann et al. (2014) inquired participants' reflections while they made self-reports of LS, PA, and NA. Participants frequently made references to life circumstances such as profession, family, and romantic relationships in relation to LS assessments, and these aspects showed a strong correlation with LS. The study also revealed that life circumstances were more prominent in participants' thoughts during LS assessments than PA and NA assessments. Additionally, participants were less fluent in expressing their thoughts during PA and NA assessments.

Instead of relying solely on verbal reports, many studies have examined the associations between measures of LS and EWB in relation to different life circumstances. In an empirical study involving samples of women from the United States and France, Kahneman et al. (2009) showed that LS had a stronger correlation with life circumstances (such as income, marital status, education, employment, living with a child, and health), while EWB had a stronger correlation with the allocation of time to various activities. It was proposed that the robust correlation between life circumstances and LS stems from individuals' attention to stable life circumstances when judging their life satisfaction, something they rarely do in their day-to-day lives. Conversely, EWB appears to depend on the outcomes of enduring circumstances in everyday life. The correlation between LS and EWB may indicate that stable life circumstances influence specific everyday conditions. An example is that wealth makes possible frequent performance of enjoyable leisure activities, while poverty instead would increase the frequency of adverse negative daily experiences.

Further support for the differences between LS and EWB arises from research indicating that EWB is more influenced than LS by time pressure in everyday life (e.g., Gärling et al., 2016; Fors Connolly et al., 2020). Schimmack et al. (2008) also highlighted that EWB has a stronger association than LS with the personality trait neuroticism, suggesting that affective dispositions have a more direct effect on EWB compared to their indirect effect on LS. Furthermore, a recent longitudinal study conducted by Kettlewell et al. (2019) revealed differences in the adjustment of LS and EWB in the short and medium term. While only partial adjustment of LS was observed within a four-year period for life events such as marriage, retirement, and childbirth, complete adjustment of EWB was for the same time interval found for the same events.

A complete understanding is lacking of why certain antecedents have different relationships with LS and EWB. People may directly consider stable life circumstances when making assessments of LS but also explicitly or implicitly place weight on other factors associated with these circumstances—factors which are more important for LS than EWB. In this registered report we propose the following four factors: social comparisons, economic and social resources, opportunities, and meaningfulness. Related to social comparisons, LS inherently carries a comparative aspect with individuals gauging their contentment in relation to others, perhaps employing a “good fortune” heuristic as suggested by Kahneman and Krueger (2006). In contrast, EWB tends to be less anchored in social comparisons, given that people's attention in everyday life is more focused on what they are doing. Thus, we hypothesize that perceiving one's life as more successful compared to others should enhance LS more than EWB (Hypothesis 1). Hypothesis 2 posits that meaningfulness correlates more robustly with LS than EWB. Despite its potential to enhance positive experiences, pursuing a meaningful life can be challenging and induce discomfort at times, tempering its positive effects on EWB (Baumeister et al., 2013). Yet, the long-term satisfaction derived from a meaningful life is likely to outweigh such emotional discomfort, thus bolstering LS.

According to Hypothesis 3, the presence of economic and social resources, both tangible and intangible (Gärling and Gamble, 2012), is more pivotal for LS than EWB. Those with resources are likely to view their lives more positively, even without immediate progress toward goals leading to subsequent increases in EWB. Economic and social resources that are critical for goal achievement increase LS, while the impact on EWB is dependent on if the resources increase goal progress (Klug and Maier, 2014). Finally, Hypothesis 4 states that opportunities are more important for LS than EWB. Both LS and EWB are related to how individuals' life is currently going, but despite experiencing temporary dips in their life individuals may report high LS if they perceive having ample opportunities of a good future life.

An illustration of how the four hypotheses may explain differences between LS and EWB observed in previous research is the following. Unemployment has in several studies been shown to be related more strongly to low LS than low EWB (Knabe et al., 2010; Schimmack et al., 2008), but it has not been clear how to explain these results. Potential explanations may be linked to the four factors hypothesized above: unemployment leads to relative deprivation, diminished future prospects, lack of meaningfulness, and limited economic and social resources (Jahoda, 1982).

For a full picture, it should furthermore be recognized that some factors may have stronger relationship with EWB than LS. Kahneman et al. (2009) suggested that individuals using their time to perform enjoyable activities often experience higher EWB due to the direct effects of these activities, while an impact on LS is less immediate (Kahneman et al., 2004, 2009; Killingsworth and Gilbert, 2010). The fifth hypothesis is that performance of recreative and enjoyable activities has a stronger relationship with EWB than LS. Hypothesis 6 posits that daily hassles and uplifts (Silva and Caetano, 2013) likewise have a stronger relationship with EWB than LS because their occurrences directly evoke affective experiences. On the other hand, LS is less sensitive to the influence of such mundane events. Finally, building on Schimmack et al. (2008), we propose that the personality trait Neuroticism is more closely related to EWB than LS (Hypothesis 7). Individuals high in Neuroticism respond stronger to negative experiences (e.g., time pressure, see Fors Connolly et al., 2020) such that EWB decreases.

In total we propose seven hypotheses (summarized in Table 1) about the relative strengths of relationships between antecedent factors and LS and EWB. To test the hypotheses, we use data from a survey of a large nationwide Swedish sample. In the questionnaire administered in the survey, conventional measures of LS and EWB are included. Novel questions are developed to measure the factors hypothesized to correlate more strongly with either LS or EWB.

**Table 1:** The hypotheses tested.

**Stronger relationship with LS than EWB**	**Stronger relationship with EWB than LS**
Hypothesis 1: Social comparisons	Hypothesis 5: Time use
Hypothesis 2: Meaningfulness	Hypothesis 6: Hassles and uplifts
Hypothesis 3a: Economic resources	Hypothesis 7: Neuroticism
Hypothesis 3b: Social resources	
Hypothesis 4: Opportunities	

LS, Life Satisfaction; EWB, Emotional Wellbeing.

To sum up, several studies have compared antecedents of LS and EWB, but they have limitations. First, they have not comprehensively examined a wide range of theoretically meaningful factors within a single study. Second, they lack preregistration, making it unclear if results were obtained through confirmatory or exploratory analyses, potentially increasing false positives and publication bias. The present study aims to address these limitations by testing a comprehensive set of preregistered hypotheses about the relative strengths of relationships between antecedent factors and LS vs. EWB, using data from a large nationwide Swedish sample.

## 2 Method

### 2.1 Participants and procedure

To examine the correlations of the hypothesized eight factors with LS and EWB, we use data from a survey of a representative sample of the Swedish population aged 15 years or older. The survey is a combined online and paper-and-pencil replication of the 11th wave of the European Social Survey (ESS) in Sweden that was conducted as face-to-face interviews. Both the sampling procedure and survey content hence closely resemble the standard ESS 11 but with an added set of questions related to SWB. As a result, many questions unrelated to the research question are included in the survey. Mirroring the approach of ESS 11, a stratified sampling technique is employed, with random samples drawn from each of the eight NUTS-2 regions in Sweden. A total of 1,500 individuals were invited to participate and offered a conditional incentive of SEK 300 in the form of gift cards, while an additional 1,500 participants were invited without being offered any incentive. The purpose of offering incentive to one group and not to the other was to investigate potential non-response bias effects. Since not directly relevant to this study, the two samples were combined in the analyses to leverage the full data set. The final net sample consisted of 1,074 individuals (35.8% response rate); however, for the analyses, we only retained participants who provided complete responses to the analyzed survey questions (n = 974). The complete stage 1 study protocol, data, and R code files can be accessed and downloaded from the OSF link: https://osf.io/4wfzv/.

### 2.2 Measures

LS was assessed using the following two items: “Imagine a ladder with steps numbered from 0 at the bottom to 10 at the top. Assume that the top of the ladder represents the best possible life for you and the bottom of the ladder represents the worst possible life for you. If the top step is 10 and the bottom step is 0, on which step of the ladder do you personally stand right now?”; and “All things considered, how satisfied are you with your life as a whole nowadays?” with end points labeled “0 Extremely dissatisfied” and “10 Extremely satisfied”. We employed merely two bipolar items to reduce the response burden while capturing evaluations that span from negative to positive wellbeing. This facilitates comparisons with EWB that likewise represents the wellbeing spectrum from negative to positive. It is consistent with Diener et al. (2012) who conceived of SWB indicators to fall on a dimension anchored at one end by judgments of satisfaction with one's life and at the other end by daily affect. Diener et al. posited that the Cantril's Ladder of Life and the single-item Life Satisfaction judgments that were used here are the measures closest to the end defined by the judgments of life satisfaction.

EWB at the other end of the continuum is assessed by means of retrospective ratings of the frequency of emotions experienced during the past week. Six unipolar adjective scales with seven steps ranging from “never” (0) to “always” (6) were used. Each scale is defined by three adjectives taken from the Swedish Core Affect Scale (SCAS) (Västfjäll et al., 2002; Västfjäll and Gärling, 2007). Participants indicated on three of the scales how often during the past week they felt positive emotions high in activation (engaged, interested, optimistic), neutral in activation (glad, pleased, happy), and low in activation (serene, calm, relaxed). On the other three scales, participants indicated how often they felt negative emotions high in activation (tense, anxious, nervous), neutral in activation (sad, displeased, depressed), and low in activation (indifferent, bored, pessimistic). We chose the SCAS as our measure of EWB because it is a validated scale in Swedish for assessing emotional experiences.

In this study self-report measures were developed of the eight candidate factors (resources are separated in Economic and Social) hypothesized to have different correlations with LS and EWB. Each were measured with two items (translated into the English) as follows:

- Social comparisons: “I am more successful than others”; and “I have achieved more than others in my age.”
- Meaningfulness: “My life is meaningful”; and “I have goals in life that are very important to me.”
- Economic resources: “My economic resources are good”; and “I have a high material living standard.”
- Social resources: “I have good support from relatives, friends, and coworkers”; and “People in my surroundings are there for me.”
- Opportunities: “I have many opportunities to do what I want in life”; and “My life is full of opportunities.”
- Time use: “I have enough time to do the things I want to do”; and “I have time for relaxing activities.”
- Hassles and uplifts: “In my everyday life, I experience too many small disturbances or obstacles”; and “There are often things that uplift my everyday life.”
- Neuroticism: “I easily feel stressed and anxious”; and “I easily feel sad and depressed.”

## 3 Results

### 3.1 Descriptive analysis

Table 2 presents the descriptive statistics of the sample (*n* = 974). The age distribution was relatively even, with the largest proportion of participants in the 55–64 age group (18.8%) and the smallest proportion in the 85+ age group (1.4%). The gender distribution showed a slightly higher proportion of women (53.2%) compared to men (46.3%). The majority of participants were born in Sweden (83.7%). The most common household income category was 72,000 SEK or more per month (15.8%), followed by 31,000–38,999 SEK (14.8%) and 57,000–71,999 SEK (11.8%). The lowest categories, up to 12,999 SEK and 13,000–15,999 SEK, had the lowest proportions of participants (3.5% and 4.2%, respectively). The largest proportion of participants were married (43.8%), followed by those who did not belong to any of the specified categories (34.8%). Widows/widowers and those in registered partnerships with deceased partners constituted the smallest proportion (4.7%).

**Table 2:** Descriptive statistics of the sample (n = 974).

**Variable**	**Category**	***n***	**%**
Age	15–24	121	12.4
	25–34	125	12.8
	35–44	126	12.9
	45–54	132	13.6
	55–64	183	18.8
	65–74	161	16.5
	75–84	108	11.1
	85+	14	1.4
	No answer	4	0.4
Gender	Man	451	46.3
	Woman	518	53.2
	No answer	5	0.5
Born in Sweden	Yes	815	83.7
	No	157	16.1
	No answer	2	0.2
Household income (SEK)	Up to 12,999	34	3.5
	13,000–15,999	41	4.2
	16,000–21,999	64	6.6
	22,000–25,999	69	7.1
	26,000–30,999	95	9.8
	31,000–38,999	144	14.8
	39,000–46,999	106	10.9
	47,000–56,999	121	12.4
	57,000–71,999	115	11.8
	72,000 or more	154	15.8
	No answer	31	3.2
Marital status	Married	427	43.8
	Registered partnership	59	6.1
	Divorced/ Registered partnership dissolved	96	9.9
	Widow/Widower/ Partner in registered partnership deceased	46	4.7
	None of these	339	34.8
	No answer	7	0.7

Table 3 gives means (M), standard deviations (SD), Cronbach's alpha (α) coefficients, and product-moment correlations for all variables. The mean scores were for LS 7.09 (SD = 1.65) and EWB 3.78 (SD = 1.00). The independent variables had means ranging from 3.06 (SD = 0.74) for Social Comparisons to 4.01 (SD = 0.67) for Social Resources. The Cronbach's alpha coefficients ranged from 0.41 for Hassles/Uplifts to 0.85 for EWB. The low alpha for the Hassles/Uplifts measure is attributed to that the scale consists of a positive statement referring to Uplifts and a negative statement referring to Hassles whose frequencies in everyday life are relatively independent of each other. LS had the strongest correlations with Opportunities (r = 0.57), Hassles/Uplifts (r = 0.52), and Economic Resources (r = 0.45). EWB showed the strongest correlations with Neuroticism (r = 0.62), Hassles/Uplifts (r = 0.52), and Opportunities (r = 0.46). Among the independent variables, the strongest correlations were observed between Opportunities and Economic Resources (r = 0.51), Hassles/Uplifts and Opportunities (r = 0.49), and Meaningfulness and Opportunities (r = 0.42).

**Table 3:** Means (M), standard deviations (SD), Cronbach's α, and product moment correlations of all variables (*n* = 974).

				**Product moment correlations**									
**Variables**	**M**	**SD**	α	**LS**	**EWB**	**SC**	**MF**	**ER**	**SR**	**OP**	**TU**	**HU**	**NE**
**Dependent variables**													
Life Satisfaction (LS)	7.09	1.65	0.81	1.00									
Emotional Wellbeing (EWB)	3.78	1.00	0.85	0.63	1.00								
**Independent variables**													
Social Comparisons (SC)	3.06	0.74	0.76	0.30	0.27	1.00							
Meaningfulness (MF)	3.76	0.72	0.59	0.38	0.33	0.38	1.00						
Economic Resources (ER)	3.47	0.79	0.63	0.45	0.33	0.41	0.26	1.00					
Social Resources (SR)	4.01	0.67	0.72	0.35	0.23	0.15	0.37	0.28	1.00				
Opportunities (OP)	3.71	0.76	0.75	0.57	0.46	0.39	0.42	0.51	0.41	1.00			
Time Use (TU)	3.67	0.87	0.79	0.30	0.32	0.01	0.12	0.23	0.24	0.33	1.00		
Hassles/Uplifts (HU)	3.23	0.68	0.41	0.52	0.52	0.23	0.35	0.34	0.32	0.49	0.41	1.00	
Neuroticism (NE)	3.07	0.96	0.82	0.39	0.62	0.20	0.16	0.26	0.11	0.32	0.25	0.40	1.00

### 3.2 Multivariate multiple regression analysis

Multivariate multiple regression (MMR) was employed to analyze the relationships between the dependent variables (LS and EWB) and the independent variables (social comparisons, meaningfulness, economic resources, social resources, opportunities, time use, hassles/uplifts, and neuroticism). MMR performs a simultaneous analysis of multiple dependent variables taking into account the correlations between them (Hartung and Knapp, 2005).

Table 4 presents the results of the MMR models of LS and EWB. Estimates of the regression coefficients, standard errors, *t*-values, and *p*-values are reported for each independent variable. In the model of LS, the regression coefficients were significant (*p* < 0.05) for the variables Meaningfulness (β = 0.104, *p* < 0.001), Economic Resources (β = 0.149, *p* < 0.001), Social Resources (β = 0.072, *p* = 0.006), Opportunities (β = 0.252, *p* < 0.001), Hassles/Uplifts (β = 0.210, *p* < 0.001), and Neuroticism (β = 0.166, *p* < 0.001). The coefficients for Social Comparisons and Time Use were not significant. Significant coefficients for EWB were obtained for Meaningfulness (β = 0.103, *p* < 0.001), Opportunities (β = 0.139, *p* < 0.001), Time Use (β = 0.055, *p* = 0.025), Hassles/Uplifts (β = 0.200, *p* < 0.001), and Neuroticism (β = 0.453, *p* < 0.001). The coefficients for Social Comparisons, Economic Resources, and Social Resources were not significant.

**Table 4:** Standardized coefficients estimated by multivariate regressions of the hypothesized independent variables on the dependent variables Life Satisfaction and Emotional Wellbeing (*n* = 974).

	**Life Satisfaction**				**Emotional Wellbeing**			
	**Estimate**	**Std. error**	***t*** **-value**	***p***	**Estimate**	**Std. error**	***t*** **-value**	***p***
Social comparisons	0.02	0.03	0.59	0.558	0.03	0.03	1.05	0.293
Meaningfulness	**0.10**	0.03	3.39	0.001	**0.11**	0.03	3.96	< 0.001
Economic resources	**0.15**	0.03	5.13	< 0.001	0.02	0.03	0.70	0.487
Social resources	**0.08**	0.03	2.89	0.004	0.00	0.03	−0.08	0.933
Opportunities	**0.25**	0.03	7.80	< 0.001	**0.14**	0.03	4.43	< 0.001
Time use	0.03	0.03	0.96	0.336	**0.06**	0.03	2.47	0.014
Hassles/uplifts	**0.21**	0.03	6.97	< 0.001	**0.20**	0.03	7.07	< 0.001
Neuroticism	**0.15**	0.03	5.84	< 0.001	**0.46**	0.02	18.43	< 0.001

Coefficients in bold are statistically significant at the 5% level.

In a test of the hypotheses (see Table 1), Table 5 displays the differences in regression coefficients between LS and EWB (LS-EWB). A positive difference indicates a stronger association with LS, a negative difference a stronger association with EWB. Neuroticism had the numerically largest difference (−0.288), followed by Economic Resources (0.129) and Opportunities (0.113). These differences were statistically significant, with Neuroticism being more strongly associated with EWB, while Economic Resources, Social Resources and Opportunities were more strongly associated with LS. Numerically smaller, non-significant differences were observed for Social Comparisons (−0.024), Meaningfulness (0.001), Time Use (−0.039), and Hassles/Uplifts (0.010).

**Table 5:** Differences between Life Satisfaction and Emotional Wellbeing (LS-EWB) in standardized regression coefficients for the independent variables (n = 974).

**Independent variable**	**LS-EWB**	**Lower 95% CI**	**Upper 95% CI**
Social comparisons	−0.01	−0.07	0.06
Meaningfulness	−0.01	−0.09	0.06
Economic resources	**0.13**	0.07	0.20
Social resources	**0.08**	0.01	0.14
Opportunities	**0.12**	0.04	0.19
Time use	−0.04	−0.10	0.02
Hassles uplifts	0.01	−0.06	0.08
Neuroticism	**−0.30**	−0.37	−0.24

Differences in bold are statistically significant at the 5% level.

### 3.3 Relative weight analysis

In addition to the MMR, a Relative Weight Analysis (RWA, Johnson, 2001; Tonidandel and LeBreton, 2015) was conducted in R using the relaimpo package (Groemping, 2006). RWA estimates the proportion of total variance in LS and EWB that each independent variable explains.

**Figure 1:**
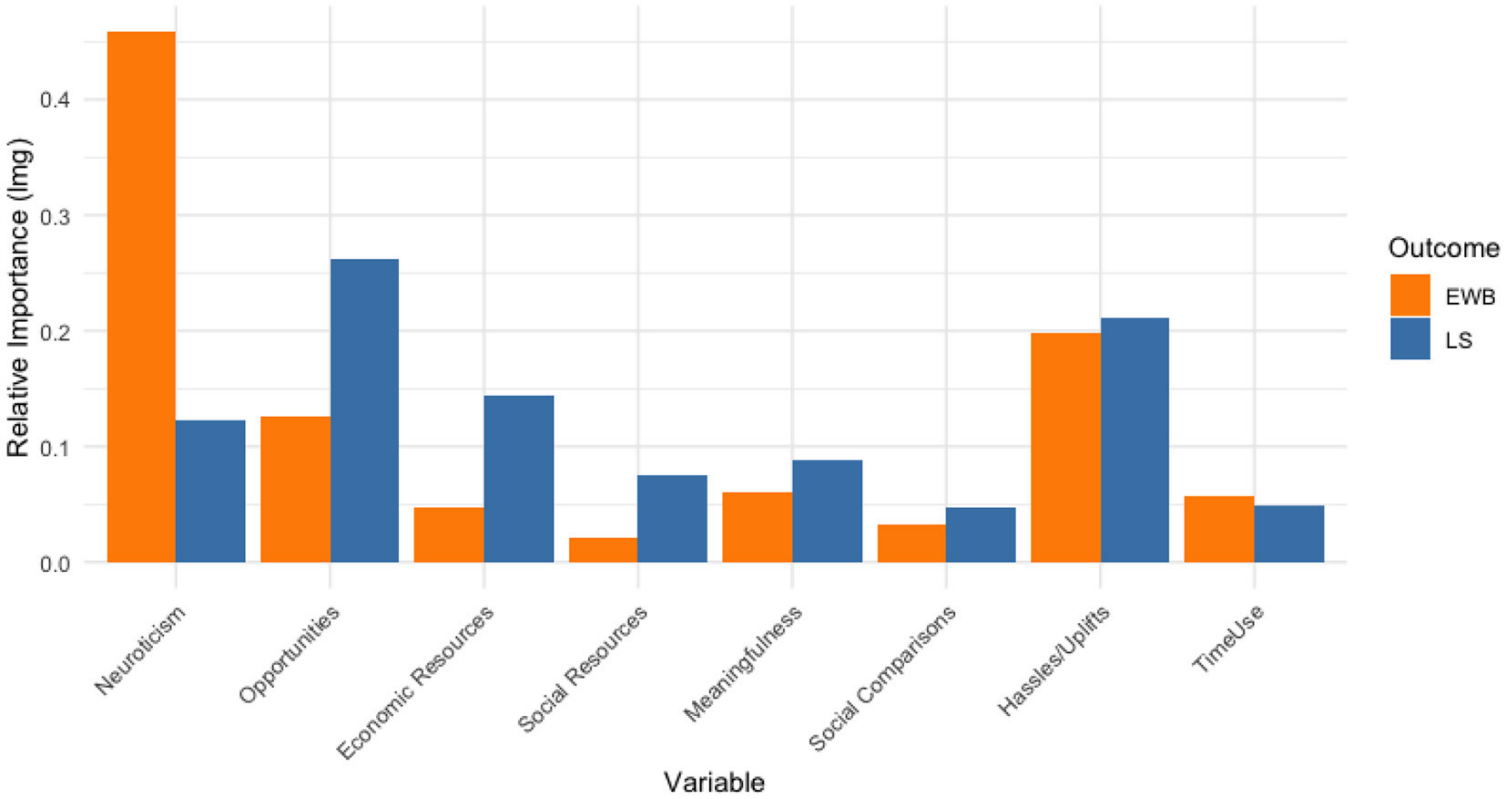
Relative importance (proportion of explained variance) of the association of the independent variables with Life Satisfaction (LS) and Emotional Wellbeing (EWB).

The results show (Figure 1) that Opportunities (0.261) and Hassles/Uplifts (0.212) explained most variance in LS, followed by Economic Resources (0.144) and Neuroticism (0.122). Neuroticism (0.458) explained most variance in EWB, followed by Hassles/Uplifts (0.198) and Opportunities (0.126). Table 6 displays the differences between LS and EWB in proportion explained variance for each independent variable. Economic Resources (0.096), Social Resources (0.055), and Opportunities (0.136) explained significantly more variance in LS than in EWB, while Neuroticism (−0.336) explained significantly more variance in EWB than in LS. The differences for Social Comparisons, Meaningfulness, Time Use, and Hassles/Uplifts were not statistically significant.

**Table 6:** Differences between Life Satisfaction (LS) and Emotional Wellbeing (EWB) (LS-EWB) in proportion explained variance estimated by relative weight analysis for each independent variable (*n* = 974).

**Independent variable**	**LS-EWB**	**Lower 95%CI**	**Upper 95%CI**
Social comparisons	0.016	−0.005	0.043
Meaningfulness	0.028	−0.012	0.067
Economic resources	**0.096**	0.057	0.140
Social resources	**0.055**	0.023	0.089
Opportunities	**0.136**	0.079	0.184
Time use	−0.008	−0.038	0.017
Hassles/uplifts	0.014	−0.042	0.071
Neuroticism	**−0.336**	−0.406	−0.268

Differences in bold are statistically significant at the 5% level.

## 4 Discussion

This study aimed at investigating the differential relations a set of antecedent factors have with LS and EWB, the two primary components of SWB. Based on previous research and theoretical considerations, we tested seven different hypothesis regarding factors that relate differentially to LS and EWB. The findings provide partial support for the hypothesized differences in the correlations between these factors and LS and EWB, respectively.

The multivariate multiple regression (MMR) analysis followed by a relative weight analysis (RWA) consistently showed that economic resources, social resources and opportunities are more strongly associated with LS than EWB, supporting Hypotheses 3a-b and 4. These results suggest that individuals' perceptions of their economic wealth, social support and future prospects play a more significant role for their life satisfaction than for their day-to-day emotional experiences. This finding is consistent with previous research highlighting the importance of stable life circumstances for LS (Kahneman et al., 2009; Luhmann et al., 2014). While previous studies have demonstrated a stronger association between income and LS than EWB, our research extends these findings by employing a broader measure of economic resources (Kahneman and Deaton, 2010). This measure captures both perceived adequacy of economic resources and material living standards. When responding, participants likely consider factors beyond just income, such as savings, assets and debt levels. Moreover, we contribute to previous studies by explicitly examining whether perceived opportunities exhibit a stronger link with LS than EWB. A moderately high correlation between perceived economic resources and opportunities (0.51) were consistent with that in the MMR analysis both factors were more closely tied to LS than EWB. This indicates that the differential relationships of opportunities to LS and EWB cannot be solely attributed to current economic resources. Opportunities may also capture expectations of future economic gains or losses, along with expectations in other domains than the personal economy.

The results provided additional consistent support for Hypothesis 3b, indicating that social resources are more important for LS than EWB. This finding suggests that the presence of supportive social networks may contribute more to individuals' life satisfaction than to their daily affective experiences, but this pattern is less pronounced than the difference in economic resources. Nevertheless, the finding is intriguing given that some previous research has pointed to the strong importance of social activities for EWB (Kahneman et al., 2009). However, our findings regarding social support can be reconciled with these results, considering that social support may act as a direct source of LS, while it may primarily affect EWB in times of need. Conversely, social activities may have a more direct influence on EWB, as being presumably more closely tied to daily affective experiences.

The results also consistenly confirmed Hypothesis 7, demonstrating that neuroticism has a stronger relationship with EWB than with LS. This finding aligns with Schimmack et al.'s (2008) argument that personality traits directly linked to affective dispositions have a more substantial impact on EWB than LS. The direct influence of neuroticism on negative affect, a key component of EWB, likely explains this differential relationship. However, it is important to recognize that there may be a conceptual overlap between neuroticism and EWB, as measures of both constructs involve affective experiences. We still believe that this finding remains meaningful, as the measure of neuroticism captures individuals' perceptions of their affective reactivity (e.g., “gets easily stressed”), while the EWB measure focuses on feelings experienced during the past week. This distinction suggests that neuroticism may influence EWB by shaping how individuals perceive and react to daily experiences, rather than only reflecting a direct overlap between the two constructs. An example is experience of time pressure (Fors Connolly et al., 2020). Furthermore, investigating the different relationships of neuroticism with LS and EWB contributes to the understanding of how personality traits relate to various aspects of wellbeing (Busseri and Erb, 2023).

Results from both analyses did not support the hypothesized stronger relationship with LS than EWB of social comparisons (Hypothesis 1) and meaningfulness (Hypothesis 2). First, social comparisons (e.g., perceiving oneself to be more successful than others) were weakly related to both LS and EWB, with associations of similar magnitude, suggesting that relative comparisons may not be that important for LS (Veenhoven and Vergunst, 2014). However, it is worth noting that the study was conducted in Sweden, which is a relatively egalitarian culture where status-seeking is less prevalent than in other countries (such as the USA, see Fors Connolly and Johansson Sevä, 2018). For this reason, social comparisons may be less important for LS in this cultural context.

A compelling case can be made that meaningfulness is more important for LS than EWB, as people can find life meaningful even during periods of distress. However, our results do not support this proposition, as the associations between meaningfulness and both LS and EWB were similar and did not differ significantly. This finding suggests that the influence of meaningfulness on wellbeing may be more complex than hypothesized, and its effects on LS and EWB more intertwined than previously thought.

The hypothesized stronger relationships of time use (Hypothesis 5) and hassles/uplifts (Hypothesis 6) with EWB compared to LS were not confirmed. These findings suggest that the influence of these factors on LS and EWB may likewise be more complex than initially assumed, warranting further investigation. We also note that the hypothesis of time use being more important for EWB than LS is primarily based on the results of studies using the Day Reconstruction Method (DRM). In the DRM (Kahneman et al., 2004), participants report time use of activities during the previous day and feelings associated with them. The average of these feelings (referred to as “net affect”) for different activities show strong differences that indirectly support the importance of time use for EWB. However, it is questionable whether the DRM captures actual experiences in an unbiased way (Lucas et al., 2021), as participants may be influenced by stereotypical notions of how activities influence EWB when assessing their feelings in connection with reported activities. Furthermore, since Luhmann et al. (2014) found that people were less fluent in reporting their thoughts when assessing PA and NA, the reliability of their findings with respect to antecedents of PA and NA may likewise be questionable. Another caveat is that direct effects of activities on EWB are not the same as long-term effects. For example, even if watching TV can be joyful for a couple of hours at night, this fact does not necessarily indicate that spending more time on TV watching is beneficial for EWB in the long run. It is understandable then that our measure of time use which targets longer-term effects (e.g., “I have enough time to do the things I want to do”) yielded different results compared to studies using the DRM. Our findings suggest that the relationship between time use and wellbeing may depend on the specific aspects of time use being assessed and the temporal scope of the measures employed. Future research should aim to clarify these distinctions and explore the potential differences between short-term and long-term effects of time use on LS and EWB.

We expected hassles/uplifts to have a stronger relationship with EWB than LS, given the direct connection to daily affective states. However, our findings suggest that the cumulative effect of daily hassles/uplifts may also have substantially association with LS. Future research should employ more fine-grained measures that distinguish between recent and long-term experiences of hassles and uplifts to better grasp the nuances between their relationships to LS and EWB.

This study contributes to the growing body of research exploring the different antecedents of LS and EWB. By identifying factors that may differentially influence these two components of SWB, the study provides a more nuanced understanding of the differences between LS and EWB. Our results may partly explain previous puzzles in the SWB research. For instance, a recent study of the unemployed (Fors Connolly and Gärling, 2022) replicated the finding that unemployment are more strongly related to LS than EWB but that financial satisfaction was only a partial mediator. Based on our results, (lack of) opportunities may be another mediator that explains why unemployment has a stronger relationship with LS than EWB. Hence, the larger association of unemployment with LS than EWB would be attributed not only to the loss of financial resources but also to the perceived lack of opportunities. This hypothesis is worth submitting to test in future studies.

The strengths of this study include the use of a large representative sample and the application of both MMR and RWA to test the hypotheses. However, some limitations should be acknowledged. First, the cross-sectional design limits the ability to draw causal inferences about these relationships. Future research employing longitudinal designs would provide further insights into their temporal dynamics. Second, the use of self-report measures may be subject to response biases, such as evaluative biases or biased recall (Schimmack, 2008). Future studies should incorporate additional methods, such as experience sampling or informant reports, to corroborate the findings. Third, our study did not explore the extent to which LS and EWB, and their relationships to antecedent factors, vary across cultural and demographic groups, as well as throughout the life cycle. This is an important nuance to consider in future studies, as it could provide valuable insights into the generalizability and contextual dependencies of our findings.

A broader potential critique of our analysis is that we did not model SWB as a general factor with LS and EWB as specific factors (i.e., a bifactor model). This approach aligns with the study of Busseri and Quoidbach (2022), who propose that SWB should be operationalized as a latent factor reflecting the commonality among momentary experiences of LS, PA and NA. While they acknowledge some unique aspects of LS, PA, and NA, they argue for a model that considers both shared and unique elements. Nevertheless, we contend that the distinction between LS and EWB (encompassing PA and NA) as an umbrella term remains justified. LS and EWB possess distinct philosophical foundations: LS is rooted in attitudinal Wellbeing theories, whereas EWB is grounded in hedonic theories of Wellbeing (Brülde, 2007). Furthermore, while the shared variance among LS, PA, and NA is empirically observable, in our assessment the notion of SWB as a *theoretically* meaningful latent construct lacks substantial support in the existing literature.

In conclusion, this study confirms that to some extent antecedent factors are differentially associated with LS and EWB, emphasizing the importance of distinguishing between these two components of SWB. The findings suggest that interventions aimed at enhancing wellbeing should consider the unique determinants of LS and EWB, and that future research should further explores the mechanisms underlying these differential relationships. Future research should also investigate potential moderators or mediators that may influence the associations between the antecedent factors and the SWB components.

## Data Availability

The datasets presented in this study can be found in online repositories. The names of the repository/repositories and accession number(s) can be found below: https://osf.io/4wfzv.
